# AB Toxins: A Paradigm Switch from Deadly to Desirable

**DOI:** 10.3390/toxins2071612

**Published:** 2010-06-25

**Authors:** Oludare Odumosu, Dequina Nicholas, Hiroshi Yano, William Langridge

**Affiliations:** 1 Center for Health Disparities and Molecular Medicine, Loma Linda University, School of Medicine, Loma Linda, CA 92354, USA; Email: oodumosu@llu.edu (O.O.); 2 Department of Biochemistry, Loma Linda University, School of Medicine, Loma Linda, CA 92354, USA; Email: dnicholas@llu.edu (D.N.); 3 Department of Biology, University of Redlands, 1200 East Colton Ave, P.O. Box 3080, Redlands, CA 92373, USA; Email: hiroshi_yano@redlands.edu (H.Y.)

**Keywords:** enterotoxins, adjuvants, AB toxins, immunomodulation, ricin, plants, vaccines

## Abstract

To ensure their survival, a number of bacterial and plant species have evolved a common strategy to capture energy from other biological systems. Being imperfect pathogens, organisms synthesizing multi-subunit AB toxins are responsible for the mortality of millions of people and animals annually. Vaccination against these organisms and their toxins has proved rather ineffective in providing long-term protection from disease. In response to the debilitating effects of AB toxins on epithelial cells of the digestive mucosa, mechanisms underlying toxin immunomodulation of immune responses have become the focus of increasing experimentation. The results of these studies reveal that AB toxins may have a beneficial application as adjuvants for the enhancement of immune protection against infection and autoimmunity. Here, we examine similarities and differences in the structure and function of bacterial and plant AB toxins that underlie their toxicity and their exceptional properties as immunomodulators for stimulating immune responses against infectious disease and for immune suppression of organ-specific autoimmunity.

## 1. Introduction

Historically, AB subunit toxins synthesized by a variety of bacterial pathogens and plants have occupied a loathsome place in man’s lexicon. They have traditionally been and still remain the subject of intense research in an ongoing effort to overcome their capacity to degrade the intestinal epithelium, resulting in severe diarrhea and the untimely death of millions of children and adults annually, largely throughout the developing world. More recently however, there has emerged a more optimistic and encouraging story suggesting that AB toxins may soon become one of man’s best allies in the battle against infection and autoimmunity. During the past two decades, AB toxins have shown increasing promise as effective, safe, and durable adjuvants for the stimulation of immunity or alternatively, the suppression of autoimmunity. In this review, we examine the similarities and differences in the structure and function of bacterial and plant AB toxins in anticipation of the scientific challenges and strategic priorities required for modern vaccine development (Table 1).

**Table 1:** Summary of Plant and Bacterial AB Toxin Structure and Function.

	A Subunit(s)	B Subunit(s)	Enzymatic Activity	Target	Receptor(s)
**Cholera toxin**	A1: 22 kDa	(×5) 10.6 kDa	ADP-ribosyl transferase	Adenylate cyclase	GM1 ganglioside
	A2: 5 kDa			*G-*protein (G_sα_)	
***E*. *coli* (LT)**	A1: 22 kDa	(×5) 11.6 kDa	ADP-ribosyl transferase	*G-*protein (G_sα_)	GM1 ganglioside
	A2: 5 kDa				Asialoganglioside
**Shiga toxin**	A1: 28 kDa	(×5) 7.7 kDa	*N-*glycosylase	rRNA (28S)	Gb3 glycolipid
	A2: 4 kDa		(Cleaves adenine 4324)		
**Pertussis toxin**	S1: 28 kDa	S2: 23 kDa	ADP-ribosyl transferase	*G-*protein (G_sα_)	GD1a ganglioside
		S3: 22 kDa			
		S4: (×2) 11.7 kDa			
		S5: 9.3 kDa			
**Anthrax**	(LF): 90 kDa	(PA): (×7) 83 kDa	Zn metalloprotease	MAPKK	ANTXR 1
	(EF): 89 kDa		Adenylate cyclase	Protein kinases	ANTXR 2
**Ricin**	30 kDa	29 kDa	*N-*glycosylase	rRNA (28S)	Glycoprotein
			(Cleaves adenine 4324)		Glycolipid

## 2. Cholera Toxin

### 2.1. Structure, Pathogenesis and Biological Function

**Figure 1:**
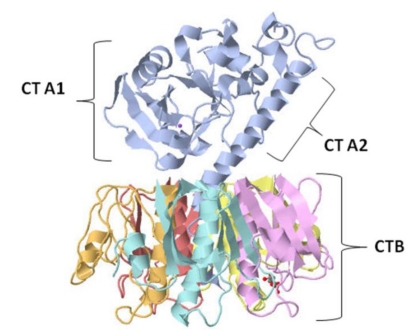
Crystal structure of cholera toxin. The heterodimeric CTA protein subunit (blue) is composed of two polypeptide chains, CTA1 (22 kDa) and CTA2 (5 kDa), linked by a single disulfide bond. The enzymatically active CTA1 peptide is the (toxic) mono-ADP-ribosyltransferase subunit, while the CTA2 helical peptide links the CTA1 subunit to the pentameric CTB subunits. The cholera toxin B subunit (10.6 kDa) is composed of five identical polypeptide subunit chains (yellow, purple, red, orange, and turquoise), each with membrane receptor GM1ganglioside binding capacity.

Cholera enterotoxin (CT) is an oligomeric protein produced in nature by the Gram negative bacterium *Vibrio cholerae*. Cholera toxin causes a high volume of secretory diarrhea initiated in the upper part of the small intestine. Initial studies suggested that the cholera toxin consisted of a single protein subunit of 84 kDa [1]. However, SDS-PAGE analysis showed CT consisted of a single large A subunit (CTA) of approximately 27 kDa and a pentameric B subunit (CTB) with an approximate monomer molecular weight (MW) of 10.6 kDa [2]. The CTA subunit was further shown to be divided into CTA1 and CTA2 subunits linked by a disulfide bond. The CTA1 subunit was found to be responsible for CT toxicity [3]. The assembly of CT structure revealed that the toxic CTA1 subunit contained ADP-ribosylating activity, while the helical CTA2 fragment was found to be responsible for embedding the CTA1 subunit into the center of the doughnut shaped CTB pentameric oligomer [4]. In addition, the CTB subunit, held together by hydrogen bonds and salt bridges, was shown to bind to ganglioside GM1[Gal(β1-3)galNac(β1-4)(NeuA-c(α2-3)Gal(β14)Glc]→ceramide [5], an anchor molecule embedded in the mammalian epidermal cell membrane [6,7] (Figure 1). Cholera toxin was shown to bind and infect a variety of somatic cells *in vivo*, especially in intestinal epithelial cells, through high affinity binding of the toxin to its cell surface receptor GM1 ganglioside [7,8,9,10]. However, only epidermal cells in the G_o_/G_1_ phase of the cell cycle were shown to both bind and internalize CT.

**Figure 2:**
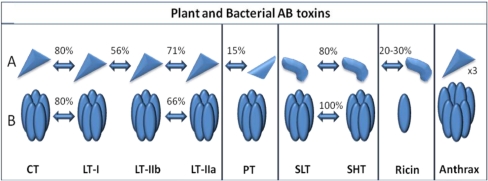
Pictorial representation of structural and amino acid sequence homologies among bacterial and plant AB enterotoxins. (**A**) The top panel represents the catalytic (toxic) A subunit proteins; (**B**) The bottom panel represents the membrane binding B subunit proteins. Approximate values for amino acid sequence homologies observed among the AB subunits depicted from different enterotoxins are provided as percentages. Enterotoxin subunits with no arrows between them share little to no amino acid or structural homologies [19].

Cholera toxin resembles another diarrhea causing bacterial toxin, the heat labile enterotoxin (LT) synthesized by the bacterial pathogen, enterotoxigenic *Escherichia coli* (ETEC). Both CT and LT share approximately 80% amino acid sequence homology and possess similar three-dimensional molecular structures with minor differences in configuration and function [4] (Figure 2). Differences between CT and LT involve proteolytic cleavage of the CTA subunit into CTA1 and CTA2, in comparison with LT, which is cleaved into LTA1 and LTA2 subunits by internal trypsinization. Moreover, CT was found to be encoded in a prophage whereas LT is encoded in a bacterial plasmid. Heat sensitive LT can bind to GM1 and GD1 ganglioside, as well as several additional intestinal glycoproteins, while CT binds preferentially and almost exclusively to the GM1 ganglioside [4,11].

Cholera toxin secretion in bacteria involves transport across the outer membrane through a CT secretion system known as the extracellular protein secretion system (Eps) [12]. The energy for secretion is provided by EpsE, a cytoplasmic ATPase that forms a complex with other secretory proteins to transfer CT across the periplasmic compartment [13]. This transfer is believed to be facilitated by the outer membrane component of the Eps, EpsD, which induces opening of the channel and subsequent secretion [14]. This protein transfer system moves CT from the periplasm, where its subunits are assembled, across the membrane, and into the extracellular environment [15,16]. In order to mediate its toxic activity, CT binds with high affinity to the GM1 ganglioside in lipid rafts on the epidermal cell surface of the lumen of the small intestine. The high binding affinity of CTB to the ganglioside GM1 is due to the contribution of a single amino acid (Gly33) on the neighboring CTB monomer to the GM1 binding site on an adjacent CTB monomer [17]. Subsequently, the crystal structure of CT revealed that Tyr12 on the CTB monomer, along with Gly33 and Trp88 on the adjacent monomer, are critical for CT-GM1 interaction [18].

The binding of CT via its CTB subunits to GM1 permits toxin endocytosis through caveolin-coated and clathrin-coated vesicles [20]. In addition, CT has been shown to enter cells through both an Arf6 dependent pathway and a non-Arf6 dependent pathway, which still remains unidentified. Interestingly, blocking all the known endocytic pathways does not appear to alter the toxicity of CT within the cell [21]. The toxin is transported not only to endosomes, but also to the endoplasmic reticulum (ER) via Golgi retrograde transport mechanisms [22]. An endoplasmic reticulum retention motif (KDEL) is located near the C terminus of the CTA chain. This motif allows the toxin to interact with the KDEL receptor, which permits the recycling of ER components from the trans-Golgi network (TGN), back to the ER [23]. Endocytosis of the toxin results in CTA1 subunit induction of adenylate cyclase. The up-regulation of adenylate cyclase activity occurs through CTA stimulation of ADP ribosylation of the adenylate cyclase Gsα subunit [24]. Increased intracellular cAMP concentrations result in an imbalance in electrolyte influx into the cell that is due to decreased sodium uptake by enterocytes and an increase in anion efflux from the cells. The decrease in sodium intake, in addition to the extrusion of anions and bicarbonates, causes water to be excreted from the cell into the lumen of the intestine. Ultimately, large amounts of water and electrolytes are lost from the intestinal epithelium, resulting in severe bouts of “rice water” diarrhea. In addition, there is fluid loss from the intestine of up to two liters per hour, leading to dehydration and death, usually by stroke in patients in which rehydration therapy is unavailable. 

### 2.2. Immunological Activity and Clinical Applications of Cholera Toxin

Enhanced immunogenic and adjuvant properties of microbial holotoxin B subunits, such as CTB and LTB, have been widely reported upon in a number of recent studies [25,26,27,28,29,30]. The intact CT holotoxin was also found to be a potent mucosal immunogen. The presence of potent CT toxic effects, CT resistance to proteases and bile salts, as well as the high affinity of CT binding to ganglioside GM1 and corresponding enzymatic ADP-ribosylating activity may contribute individually or together toward establishing the powerful immunostimulatory activity of CT [11]. Despite its strong immunogenic activity, and its wide use in animal vaccination protocols, the ability of CT to induce persistent inflammation has delayed application of the holotoxin as an adjuvant for stimulating immune responses in human vaccines. 

Despite this present drawback, oral CTB cholera vaccines have proved to be effective against cholera toxin [31]. Surprisingly, in addition to its known capacity to induce a pro-inflammatory response, oral administration of CTB subunit, when coupled with an autoantigen, was shown to induce a state of immunological tolerance [32,33]. In early studies, oral delivery of CTB conjugated to specific autoantigens was shown to enhance autoantigen mediated protection of mice against several autoimmune diseases, including autoimmune encephalomyelitis [29], autoimmune chondritis [34], and uveitis [35]. Further, CTB-autoantigen conjugates were shown to substantially suppress Type 1 autoimmune diabetes in non-obese diabetic (NOD) mice [36,37]. The results of the diabetes studies indicated that CTB-autoantigen conjugates reduced IFN-γ production and the migration of Tr1 regulatory T cells into pancreatic islets [38,39]. Linkage of CTB to an autoantigen was shown to provide up to a 10,000 fold reduction in the amount of autoantigen required for generating immuno-tolerance [32,40].

Mechanisms underlying CTB-autoantigen activated immunological tolerance were shown to include inhibition of dendritic cell (DC) maturation, autoreactive T cell development or stimulation of Th2 and Foxp3 regulatory T cell proliferation and activation [41,42,43], or both. In other studies, incubation of immature DCs with CTB was shown to induce DC maturation in experimental tumor models [32,44]. Morphological changes in DCs incubated with CTB included cell enlargement, elongation of DC dendrites, and increased migration of DCs into draining lymph nodes, as well as increased expression of the B7-2/CD86 co-stimulatory molecule [44]. Further, the mucosal administration of CTB conjugated to autoantigens was shown to mediate synthesis of T cell cytokines in response to the antigen or autoantigen complex. In experimental allergic encephalitis studies, the secretion of proinflammatory cytokines IL-12, IFN-γ, and TNF-α were significantly reduced while T cell expression of TGF-β was increased in animals treated with CTB conjugated to myelin basic protein (MBP) [29]. Similarly, immunosuppressive cytokine secretion, including increased IL-10 secretion, was observed after oral administration of CTB conjugated to insulin, resulting in suppression of diabetes onset in NOD mice [36,38,45]. Based on the recent findings that inflammatory TH17 lymphocytes were implicated in autoimmune disease pathogenesis, conjugation of CTB to myelin oligodendrocyte glycoprotein (MOG), delivered together with Complete Freund’s Adjuvant (CFA), resulted in suppression of lymphocyte IL-17 secretion [46]. New findings from our laboratory show that a fusion gene encoding the cholera toxin B subunit (CTB) linked to the diabetes autoantigen glutamic acid decarboxylase (GAD), when delivered by recombinant vaccinia virus inoculation in combination with small amounts of IL-10 or complete Freund’s adjuvant (CFA), generates effective and durable suppression of autoimmune type 1 diabetes insulitis and hyperglycemia in non-obese diabetic (NOD) mice [47]. This combinatorial vaccine approach is able to completely suppress autoimmune diabetes onset without subjecting the patient to significant levels of systemic IL-10 or CFA toxicity. These findings establish a solid basis for clinical assessment of vaccine efficacy in early onset diabetes patients and in those prospective patients who are genetically predisposed to development of insulin dependent diabetes mellitus (Type 1 diabetes).

## 3. Heat Labile Enterotoxin from Enterotoxigenic *E. coli* (LT)

### 3.1. Structure, Pathogenesis and Function

**Figure 3:**
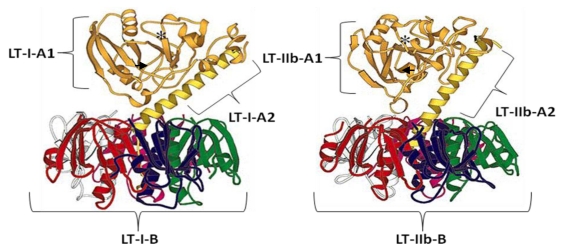
The crystal structures of Heat Labile *E*. *coli* LT-1(Left) and LT-IIb (Right). The A1 subunits are shown in gold and the A2 subunits in yellow. The individual B subunits are displayed as red, white, pink, green, and blue ribbon structures. The region containing the active-site of each molecule is highlighted by an asterisk and the disulfide bond in the A subunit is indicated by black arrows. The structure of Heat Labile *E*. *coli* LT is adapted from Focco van den Akker *et al*. 1996 [49].

The Heat labile toxins (LT), produced by enterotoxic strains of *E*. *coli*, can be categorized into type I (LT-I from now on referred to as LT) and type II (LT-IIa and LT-IIb). Both toxin types are structurally and functionally similar to Cholera toxin (CT). As previously stated, LT exhibits extensive amino acid homology to CT, while the A subunits of the type II counterparts exhibit amino acid homology to LT-I (Figure 2). Like CT, LT is an AB toxin, with one catalytic A subunit (LTA), which binds to a pentamer of non-toxic B subunits (LTB). Interestingly, the first crystal structure of a complete hexameric AB toxin was that of LT in 1991 [48]. This crystal structure shows that LTA (27 kDa) is composed of two subunits joined by a single disulfide bridge: A1, the active catalytic portion, and A2, a linker peptide that attaches the A1 subunit to the LTB pentamer (Figure 3).

The secondary structure of LTB plays a role in its function and pentameric subunit association. The *N-*terminus of LTB is an α-helical loop (α1 helix) and has been implicated in various roles in the function of LTB. The *N-*terminal α1 helix is located outside the core structure and is linked by disulfide bonds. The *N-*terminus has been implicated in stabilizing the toxin protein by serving as protection against proteolytic degradation [50]. In addition, the GM1 binding site for the holotoxin is located near the *N-*terminus. Deletions in the LTB subunit protein α1 helix, which affect the secondary structure, reduce the binding affinity of the B subunit for its GM1 receptor. In addition, the α1 helix mutants, ΔQ3 and E7G, greatly curtail LTB secretion [51]. Most interestingly, the *N-*terminal decapeptide region of each individual subunit has been found necessary for pentamer formation, as noted by the inhibition of complex formation observed by antibody blocking of this region [52].

The pentameric complex of LTB monomers (11.6 kDa) forms a ring structure with a pore having a length of 30 Å and an average diameter of 13 Å [48]. This structure acts as the delivery system for the LTA subunit (27 kDa), via interaction with ganglioside membrane receptors on the host cell. Previous experiments have shown that LTB has a strong interaction with the ganglioside receptor GM1, which is partially facilitated through the free carboxyl of sialic acid on the receptor, as noted by detection of a reduction in LTB binding affinity to asialic GM1 [53]. LTB interacts directly with lactose, and exhibits high promiscuity in receptor binding. In addition to the GM1 receptor, LTB also interacts with paraglobosides, GM2, polyglycoceramides, and polylactosamine-containing glycoproteins, although with lower affinity [53,54,55,56,57]. Both LT-IIa and LT-IIb are even more deviant in their receptor binding specificities. LT-IIa was shown to bind preferentially to ganglioside GD1b, although it may also bind GD1a and GM1 with lower avidity [58]. Finally, LT-IIb is known to bind only to receptor GD1d [58]. 

The simultaneous binding of pentameric LTB to five GM1 ganglioside receptors initiates the toxin’s uptake into the cell [54]. The process of toxin endocytosis and translocation to the cytoplasm is essential for toxin function. The initiation of toxin endocytosis is connected to the association of GM1 receptors with lipid rafts embedded in the cell membrane. This localization to lipid rafts is deemed necessary for complex uptake [59]. As observed with CT, toxin complex endocytosis may be mediated through a variety of uptake pathways including clathrin-dependent, caveolar-dependent pathways, or potentially by mechanisms independent of either. The toxin is then trafficked in a retrograde manner, eventually reaching the endoplasmic reticulum (ER). From the ER, it is believed that oligosaccharide specific sorting allows the LT A1 subunit to be translocated to the cytoplasm, rather than via a protein specific pathway [20]. Once in the cytoplasm, disulfide bonds linking LTA1 and LTA2 are reduced, liberating the active toxic A1 subunit. Similar to CTA, The catalytic A1 subunit of LT acts as an ADP-ribosyltransferase that activates the G protein G_sα_. This G protein activation causes cytotoxity via the activation of adenylate cyclase, which then increases intracellular levels of cAMP [60]. The increased levels of cAMP subsequently causes the well-known pathogenesis of “rice water” diarrhea and dehydration characteristic of infected hosts.

### 3.2. Immunological Activity and Clinical Applications of LT

LT interacts with the immune system in various capacities. Fascinatingly, these interactions, which are still being characterized, provide LT and more specifically, LTB, particular immunomodulatory functions. This enterotoxin and its nontoxic B subunit were shown to possess strong adjuvant properties that magnify immune responses towards co-delivered or conjugated antigens. Due to the possible LT holotoxin neurotoxicity observed in Bell’s palsy correlation studies, the LTB subunit, rather than the holotoxin, should be considered as the dominant focus for future clinical applications [61]. Counter-intuitively, it seems that LT and LTB have the potential for stimulation of either pro-inflammatory or anti-inflammatory immune responses, depending on the nature of the linked antigen. Thus, the relationship of LTB to future clinical applications may be based on the manifestation of these two capacities.

The *E*. *coli* heat sensitive holotoxin LT, and its toxin B subunit, LTB, may induce immunity by different mechanisms. Epidermal-like CaCo-2 cells incubated with LT were shown to inhibit IL-2 cytokine secretion by T lymphocytes [62]. Further, the antigen presenting capacity of B cells *in vitro* is increased by catalytic LT [63]. The ability of LTB and LT to retain adjuvant capacity is indicative of the toxin’s interaction with other cell types. The B subunits of LT-IIa and LT-IIb induce the production of IL-8, IL-6, IL-1β, and TNF-α by monocytes [64]. LTB also has the ability to induce high levels of TNF-α production by murine macrophages [65]. The other cell type of major interest is the dendritic cell, which is thought to be an arena for toxin subunit adjuvant activity. Incubation of immature DCs with LT-IIb-B induces TLR-2 dependent activation, as marked by DC costimulatory factor CD86, CD80, and CD40 upregulation [66]. These activated DCs have the capacity to increase CD4+ T cell proliferation [67]. Adjuvant potency is further realized by LT’s ability to mediate DC migration. Experiments have shown that LT induces a localization of DCs to the follicle-associated epithelium of the Peyer’s patches [68]. This result may help to explain the observed increase of antigen uptake into DCs associated with adjuvant/antigen co-delivery. 

The practical applications of mucosal subunit vaccines are becoming increasingly apparent. The use of LTB as an adjuvant molecule in the development of vaccines against a variety of diseases has recently been assessed. In conjunction with viral vaccines, LTB was used to increase immune responses and serum antibody titers. Administration of the influenza vaccine A/H5N1 with an adjuvant LT patch at the delivery site was shown to be safe. The treatment conferred greater serum antibody titers in patients [69]. This observation opens up the possibility of using LTB enterotoxin B subunit adjuvants to enhance inflammatory immune responses for other viruses, such as HIV [70]. In addition to preventive therapy for viral infection, LTB conjugates have demonstrated the ability to confer protection against bacterial infections. LTB fused with several T and B cell epitopes from *H*. *pylori,* and delivered orally as a vaccine into mice, decreased bacterial colonization upon *H. pylori* challenge [71]. Further advancement in the field of subunit vaccination can be seen in the use of LTB as an adjuvant in the prevention and treatment of cancer and neurodegenerative diseases. For example, LTB-CEA (carcino-embryonic antigen) fusion protein exhibit antitumor protective effects when administered before a tumor challenge [72]. Even more promising are the current clinical trials, in which a mutant LT (R192G) adjuvant is co-delivered with peptides from amyloid-beta for the treatment of Alzheimer’s disease [73]. 

Another major area of LTB and some LT mutant adjuvant development involves immune tolerance and modulation of the immune system towards an anti-inflammatory state. This type of adjuvant activity is directly applicable to autoimmune disease. Specifically, LTR72, a partially detoxified mutant, was shown to inhibit development of TH1 cells and to augment the activation of TH2 cells *in vitro* [74]. In mouse models, LTB provided protection from both autoimmune uveoretinitis and encephalitis via co-administration with and direct linkage to the auto-antigen, respectively [75,76]. Our laboratory has shown that administration of LTB adjuvant protein fused to glutamic acid decarboxylase (GAD), as well as CTB-GAD and STB-GAD, is able to mediate suppression of Type 1 diabetes development in NOD mice [77]. A current interest is in establishing a method for oral administration of subunit vaccines in geographic areas with less or no present access to healthcare. Edible plants transformed with genes encoding the desired adjuvant and antigen fusion protein present an ideal route. The expression of various vaccine combinations linked genetically to LTB as an adjuvant have been synthesized in edible plants, including potatoes, carrots, lettuce, rice, and corn [78,79,80,81,82]. These plant production and delivery vehicles could provide an optimum route for exploiting the adjuvant potential of bacterial enterotoxins.

## 4. Shiga and Shiga-like Toxins

### 4.1. Structure Pathogenesis and Function

For many years, Shiga toxin (named after Kiyoshi Shiga, a Japanese bacteriologist, who first identified *Shigella* *dysenteriae* as the cause of inflammatory dysentery diarrhea), was thought to be exclusively produced by *Shigella* bacteria [83]. However, it is now known as a toxin family that includes Shiga toxin and other closely related toxins. Shiga toxin (Stx) is produced by *S.* *dysenteriae*, while Shiga-like toxin-1 (Stx1) and Shiga-like toxin-2 (Stx2) are produced by Enterohemorrhagic *Escherichia coli* (EHEC) or Shiga toxin producing *E*. *coli* (ETEC) [84,85,86]. The infamous *E*. *coli* strain O157:H7 that causes severe human diarrhea or dysentery belongs to the type of *E*. *coli* that produces Stx2 [85]. Common characteristics shared among these bacterial strains are that they are Gram-negative, rod-shaped, anaerobic, and non-sporulating [84]. 

**Figure 4:**
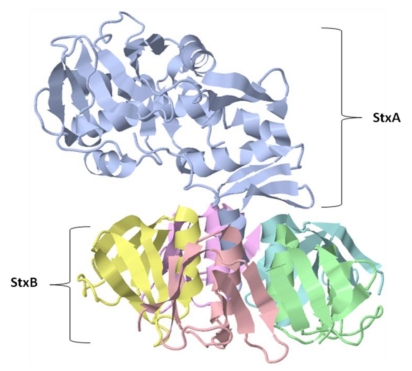
The crystal structure of Shiga toxin (Stx) protein. The A subunit StxA (blue), is composed of two peptide fragments covalently associated by a single disulfide bond: A1 (28 kDa) and A2 (4 kDa) [26,31]. Cleavage in the Golgi apparatus of these two fragments at Cys242–Cys261 is required for the activation of the A1 fragment [20,26]. The individual StxB subunits associate to form a homopentamer, with each individual StxB subunit possessing a molecular weight of 7.7 kDa [27].

Shiga toxin is a hexameric protein with a molecular mass of 70.5 kDa. It is composed of an enzymatic subunit StxA monomer and a receptor-binding StxB homopentamer (Figure 4). While StxA is 32 kDa, each StxB subunit of the pentamer is 7.7 kDa [87]. Within the StxA subunit, there are two fragments covalently associated by a single disulfide bridge: A1 (28 kDa) and A2 (4 kDa) [85,88]. How those subunits associate to form the holotoxin is shown in Figure 4. The *C-*terminus of the A2 fragment is responsible for AB subunit association: the Leu282, Gly283, Ala284, Ile285, Leu286, and Met287 residues that partially penetrate the StxB subunit [89] are especially involved. The toxic A1 fragment has significant amino acid sequence homology to the A subunit of the plant enterotoxin, ricin, and is similar to it at both structural and functional levels (Figure 2) [89,90]. The Stx A1 fragment and ricin toxin both depurinate adenosine in the 28S ribosomal RNA of the 60S ribosomal subunit. This modification makes tRNAs that are unable to associate with the ribosomal complex, which ultimately leads to the inhibition of protein synthesis in target cells [85,86,89,90]. When dissociation of tRNA from the ribosome occurs, JUN *N-*terminal kinase (JNK) and mitogen activated protein kinase (MAPK) p38 are activated, leading to an alternation of extracellular signal-regulated kinase (ERK)-1 and ERK-2 signaling pathways [85]. When epithelial cells are targeted, the p38-dependent pathway induces IL-8 secretion, and when the target cell is a monocyte, GM-CSF and TNF-α secretion are induced in a p38 pathway dependent manner [85]. However, in order to fully obtain enzymatic activity of the A1 fragment, the StxA subunit must be proteolytically cleaved in the Golgi cisternae at the A1-A2 disulfide bridge [89]. 

The StxB subunit is a symmetric homopentameric ring composed of five identical B subunits. However, despite its symmetric structure, StxB associates with StxA asymmetrically by having only three of its B subunits interacting with the *C-*terminus of the A2 fragment, thus making StxA bend to the side opposite from the three B subunits (Figure 4) [89]. This conformation is seen in the B subunits of other AB toxins, which bind to specific receptors with specific glycolipids or glycoproteins. StxB preferentially binds to globotrioylceramide (Gb3)and facilitates the internalization of StxA into the target cell [85,89]. However, it has been found recently that StxB, which was believed to be the non-toxic subunit of Stx, actually has significant toxic activity in the target cell. The toxicity of the StxB subunit consists of DNA cleavage that leads to apoptotic cell death [90]. Apoptotic cell death appears to occur via a caspase 8/3 or 8/3/6 cascade pathway, in addition to decreased expression of survival factor B-cell lymphoma-2 (Bcl-2) and an increase of the TNF-related apoptosis-inducing ligand (TRAIL), followed by Ca^2+^ release from the ER [85].

The cell entry mechanism for Shiga toxin proteins is via a retrograde transport system, which was first elucidated by a study focused on Stx entry into cells [90,91]. Stx binds to Gb3 ganglioside in lipid rafts on the target cell membrane and initiates endocytosis. Stx is then carried into the trans-Golgi network (TGN) through the perinuclear endocytic recycling compartment (ERC) by clathrin-coated vesicles. From the TGN, Stx travels to the ER in coat protein complex I (COPI)-coated vesicles. However, unlike other AB toxins, such as cholera toxin, that depend on KDEL (a lys-asp-glu-leu amino acid sequence) to facilitate retrograde transport to the ER, Stx appears to remain KDEL-independent [86,90]. Once Stx enters the ER lumen, it links with a pre-associated large multi-chaperone complex of HEDJ, BiP, and a 94 kDa glucose-regulated protein (GRP94), in order to partially unfold so that the ER can recognize it as a misfolded protein and excrete it into the cytosol via ERAD. Typically, misfolded proteins are degraded by the proteosome. However, Stx lacks lysine residues and cannot be ubiquitinated. Therefore, proteosomes do not see the toxin as a target and Stx maintains its enzymatic activity in cytosol of the target cells [85,86,90]. The proteolytic cleavage of StxA at Cys242–Cys261 for the activation of the toxic A1 fragment is accomplished in vesicles of the Golgi apparatus by a proteolytic enzyme furin, along with glycosylation in the ER during retrograde transport from the Golgi to the ER [85,90].

### 4.2. Immunological Activity and Clinical Applications of Shiga Toxin

From extensive studies of Stx functions, it is known that the toxin interacts with antigen presenting cells (APC), predominantly dendritic cells and macrophages, and induces APC expression of costimulatory factors CD40, 80, 83, and 86 and major histocompatibility complex (MHC) classes I and II [90]. In addition, Stx is known to induce inflammatory cytokine IL-1, IL-6, and TNF-α secretion by macrophages [86]. At present, the type of immune response that Stx induces downstream of those antigen presenting cells remains inconclusive. Haicheur *et al*. demonstrated that when splenocytes stimulated with StxB-OVA recombinant protein were co-incubated with OVA specific CD4^+^ T cells, both IL-2 and IFN-γ secretions were enhanced, indicating that a Th1-type response was induced by the StxB subunit [92]. Additionally, Ohmura *et al*. showed that bone marrow derived DCs (BMDCs) incubated with either Stx1 or its B subunit (StxB1) differentially induce Th1-, Th2-, and possibly Th17-type responses, as demonstrated by the types of cytokines secreted [87,93]. Further, the same authors found that BMDCs incubated with StxB1 induced secretion of TNF-α and IL-12p70. When BMDCs stimulated with Stx1 were co-incubated with CD4^+^ T cells, secretion of IL-4, IL-5, IL-6, IL-10, and INF-γ cytokines was induced. However, when BMDCs stimulated with StxB1 were co-incubated with CD4^+^ T cells, only IL-6 secretion was significantly enhanced [93]. These results confirm that Stx1 is capable of inducing both Th1 and Th2-type responses [93]. Also, StxB1 appears to skew the T cell population towards an inflammatory Th17 phenotype, as IL-6 is one of the early cytokines secreted by Stx inoculated DCs, and is essential for Th17 cell differentiation [87]. In addition, cytokines induced by Stx, especially IL-1 and TNF-α, can induce synthesis of Gb3, which attracts the binding of additional Stx molecules. Thus, a positive feedback loop for increasing target cell sensitivity may be a possibility [86].

Based on the internalization mechanism of StxA subunits, recombinant protein vaccines have been designed that deliver antigen epitopes into the ER lumen for presentation to T cells by MHC class I receptors that present them on the cell surface [86,90]. Antigen presentation induces in turn the CD8^+^ cytotoxic T cell responses [85,94]. Further, as demonstrated by Adotevi *et al*., co-administration of StxB-antigen recombinant protein with α-galactosylceramide (α-GalCer) increases the efficiency of antigen delivery during StxB-mediated internalization. This approach required only 50 ng of immunogen dosage to induce a CD8^+^ cytotoxic T cell response [94]. Most importantly, when transgenic mice that produce OVA were co-stimulated with StxB-OVA and α-GalCer, CD8^+^ T cells specifically active against OVA were detected. This result suggests that StxB recombinant protein delivered with α-GalCer may induce immune responses to microbial diseases, and also against tumors that require recognition of self-antigens, through modulation of the immune system by immunogenic proteins fused to the StxB subunit [85,94].

In another example of StxB recombinant vaccines, Oloomi *et al*. showed that by immunizing mice with a fusion protein encoding aggregative adherence fimbria (StxB-AAF), a humoral immune response was mounted against bacteria that use AAF to colonize epithelial cells. In addition, immunity against a lethal dose of Shiga toxin was also generated [95]. Because there are no vaccines against Shiga toxin producing bacteria established to date, this technique could be useful for arresting disease onset by inhibiting the colonization and progression of bacterial invasion through induction of a humoral immune response specifically against Shiga toxin [95].

The StxA subunit can also be used as an adjuvant in recombinant StxA subunit vaccines, which may target specific cells, depending on what kind of protein is fused to StxA [85,90]. One example of this type of subunit vaccine is the recombinant protein StxA-CD4. The acquired immune deficiency syndrome (AIDS) causing virus HIV-1 uses its coat glycoprotein, gp120, to bind CD4 on the surface of leukocytes, including T helper cells and dendritic cells. These infected immune cells present gp120 on their cell surface. By fusing StxA with CD4 cell derivative peptides, StxA toxicity may be delivered specifically to infected cells without incurring damage to healthy uninfected cells [70,96]. In all, the use of STX and its subunits as adjuvants for the induction of immunity is becoming more defined.

## 5. Pertussis Toxin

### 5.1. Structure, Pathogenesis and Function

Pertussis Toxin (PT), a protein synthesized by the Gram-negative coccobacillus *Bordetella pertussis*, is primarily toxic to epithelial cells of the respiratory tract [97,98]. *B*. *pertussis* is a strict human pathogen known to be responsible for Whooping Cough, a highly contagious childhood respiratory disease named after the unusual low-pitched, distinctive repetitive cough expressed by infected patients. The bacterial pathogen responsible for Whooping Cough was first identified as *Bordetella pertussis* by the Belgian scientist, Jules Bordet, in 1906 [99]. Because early pertussis vaccines were constructed from attenuated bacteria, there was a concern that immunized adults may double as carriers of the pathogen and be responsible for the infection of new born infants not yet immunized [84]. However, in the United States, an acellular diphtheria, tetanus, and acellular pertussis “toxoid” vaccine (DTaP) was adopted by the Center for Disease Control (CDC) to reduce the opportunity for infection of infants [100]. 

**Figure 5:**
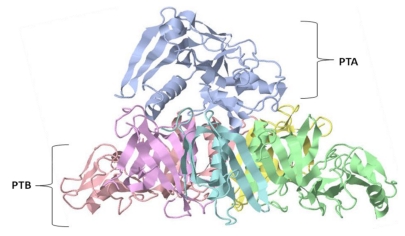
The crystal structure of pertussis toxin (PT). The PT protein subunits associate in a hexameric configuration with a combined molecular weight of 105 kDa. The PT subunits; S1 (blue), S2 (green), S3 (pink), S4 (yellow and purple), and S5 (turquoise); associate in the ratio 1:1:1:2:1. The S1 subunit is pyramidal and is composed of the A subunit (PTA) with a molecular weight of 28 kDa [9,30]. The PTB subunit is composed of S2 (23 kDa), S3 (22 kDa), two S4’s (11.7 kDa each), and an S5 (9.3 kDa) oligomer [30,32], creating an asymmetrical heteropentameric ring structure [2,6,9,11].

Pertussis toxin has a six membered oligomeric structure (Figure 5). The single pertussis A subunit displays enzymatic activity that transfers ADP-ribose from nicotinamide adenine dinucleotide (NAD) to the cysteine residue of trimeric guanine nucleotide-binding proteins (*G-*proteins), leading to a decoupling of the *G-*protein α-subunit from its receptor. Subsequently, this decoupling event prevents the inhibition of adenylate cyclase activity, resulting in an increase of intracellular cAMP concentration [97,101,102]. Other AB toxin A subunits, for example the A subunit of the heat-labile enterotoxin (LT) from *Escherichia coli*, share a similar function, with the exception that the LTA subunit protein attacks an arginine residue in *G-*proteins rather than a cysteine residue [97]. In contrast to the toxic enzymatic function of the A subunit, the pertussis B subunit (PTB) binds preferentially to receptor glycoproteins and glycolipids, such as ganglioside GD1a, which is located within lipid rafts embedded in the host cell plasma membrane [97,103]. The binding of PTB to its receptor is believed to initiate retrograde transport of the pertussis A subunit (PTA) or PTB through the *trans*-Golgi Network (TGN) to the endoplasmic reticulum (ER), or both [23,98,101,104]. As PTA travels through the TGN, it undergoes tyrosine sulfation and *N-*glycosylation in the Golgi apparatus and ER, respectively, in order to fully establish ADP-ribosylating ability [97]. In addition, in a manner similar to other toxins like cholera toxin (CT) and Shiga toxin (Stx), PTA is believed to be partially unfolded in the ER lumen so that the ER recognizes it as a misfolded protein and excretes it into the cytosol for endoplasmic reticulum associated protein degradation (ERAD) [23,101]. Just like StxA, PTA lacks lysine residues and cannot be ubiquinated. Therefore, ERAD cannot recognize PTA as a target. Thus, PTA evades degradation in the proteosome and can initiate its toxic enzymatic function [23,102].

The majority of the pertussis toxin AB subunit associating region is the flat basal portion of a pyramidal PTA subunit sitting on an asymmetric heteropentameric ring of the PTB subunits. At the center of this PTB ring, five helices create a hole where short extended strands of PTA (β10, 11, and 12) partially penetrate [97]. Unlike the majority of AB toxins, PT does not require proteolysis for activation of its A subunit. However, reduction of the disulfide bond between Cys41 and Cys201 is essential for enzymatic function. This observation is consistent with the idea that a region near Cys201 must shift in order for NAD and *G-*protein to have access to the active site of PTA. Within the active site, Glu129, Cys 41, and Trp26 are believed to interact with NAD and Arg9. In addition, Asp11, Arg13, and Phe23 are also important for maintenance of conformation of the toxin’s active site [97].

Within PTB, five subunits (S2, S3, two S4s, and S5) are associated into three distinctive groups: S2/S4 dimer, S3/S4 dimer, and S5 monomer. Both S2 and S3 are known to be 70% identical in amino acid sequence, and the Cys27–Cys83 region has significant homology to rat mannose binding protein (MBP), which belongs to the *C-*type lectin family. This homologous region, based on lectin’s carbohydrate recognition domain (CaRD), is believed to provide PTB’s sugar specific binding for adherence to the host cell membrane [97]. Furthermore, the properties of S2 and S3 subunit penetration of host cell lipid rafts may be involved in facilitation of PTA (S1 subunit) internalization [105].

### 5.2. Immunological Activity and Clinical Applications of Pertussis Toxin

Interestingly, although PTA and LTA share extensive amino acid sequence homology, the conformation of their B subunits is completely different. As seen in Figure 2, PTA and LTA associations to B subunits are rotated 180°, which results in a detectable difference in the active site conformation that, in turn, results in a difference in the target residues: cysteine (PT) and arginine (LT) [97]. This difference in structure provides the basis for PT and PTB’s alternate interaction with the immune system.

Both PT and PTB differentially activate dendritic cells (DCs) by stimulating the Toll-like membrane receptor (TLR)-4 [106]. The pertussis toxin B subunit preferentially triggers activation of the adaptor protein myeloid differentiation primary response gene (MyD88)-independent pathway, leading to induction of chemokine ligand (CXCL or IP)-10. Thus, PT can trigger both MyD88-independent and MyD88-dependent pathways. Subsequently, PT induces not only IP-10, but also pro-inflammatory cytokines IL-6, TNF-α, and IL-12 [106]. Fujimoto *et al*. and Hou *et al*. demonstrated that DCs stimulated with PT upregulate cytokines IL-12, IL-6, IL-1β, IFN-γ, and TNF-α, resulting in only small amounts of IL-10 and almost no IL-4 secretion. Under these conditions Th1 inflammatory immune responses are favored [107,108].

As previously demonstrated by Chen *et al*., PT stimulation of CD4^+^CD25^+^ T cells suppressed both Foxp3^+^ expression and IL-2 secretion. Further, it was found that PT also decreased the number and function of lymphocytes that suppress CD4^+^CD25^-^ T cells. Because IL-2 is one of the critical cytokines that support T regulatory (Treg) cell differentiation and proliferation, the immune system could, in general, be skewed towards a more inflammatory condition due to decreased Treg cell availability [109]. 

Based on the ability of PT to stimulate a Th1 lymphocyte favored environment upon interaction with dendritic cells and T cells, PT has been extensively studied for its immunological adjuvanticity. However, due to the strong toxicity of the PTA subunit, the non-toxic PTB subunit is favored for use as an adjuvant [109]. Immunization experiments with PT-adjuvant fusion proteins were shown to suppress Th1-mediated organ specific autoimmune diseases, including experimental autoimmune encephalomyelitis (EAE) and experimental autoimmune uveitis [70,109]. As observed with complete Freund’s adjuvant (CFA), PT can increase the permeability of the blood-brain barrier (BBB) to leukocytes. With its ability to modify BBB permeability, PT was shown to enhance a CD4^+^ Th1 cell-mediated inflammatory response in the central nervous system (CNS) when EAE-susceptible mice were co-immunized with myelin basic protein, CFA, and PT [109].

In addition to its adjuvanticity, PT has shown potential for fighting human immunodeficiency virus type 1 (HIV-1) infection. Pertussis toxin was found to inhibit HIV-1 infection in two ways: both pre- and post-entry of the virus into host cells [70,110]. In the pre-entry stage, PT, especially PTB, can inhibit R5 HIV-1 entry to primary CD4^+^ T cells via densitization of CC chemokine receptor type (CCR)-5, leading to uncoupling of CCR-5 from CD4, a primary receptor for HIV [70,110]. Secondarily, PTB can also inhibit HIV-1 infection through down-modulation of HIV replication. Both Tat-dependent HIV transcription and the stability of subsequent HIV mRNA products are inhibited by PTB [70,111]. PTB is related to post-entry HIV-infection inhibition and can thus also interfere with latent HIV-1 multiplication in chronically infected cells through the Activator Protein 1 (AP-1) dependent pathway [112]. These observations reveal the increasing need for research involving the use of PTB as an adjuvant.

## 6. Anthrax Toxin

### 6.1. Structure, Pathogenesis and Function

Anthrax is an AB enterotoxin produced by the Gram positive bacteria, *Bacillus anthracis*. Unlike other AB toxins described thus far in this review, anthrax toxin has a tripartite structure, consisting of three independent polypeptide chains. These three subunits are denoted as edema factor (EF), lethal factor (LF)—both of which have enzymatic activity—and protective antigen (PA). The cytotoxic and immunogenic functions of anthrax are coupled to these subunits’ molecular structures and their interactions.

**Figure 6:**
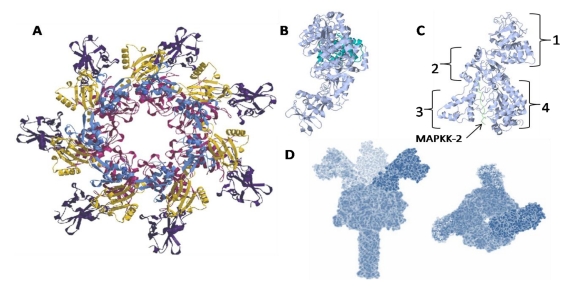
Computer modeled top-down view of anthrax toxin protective antigen (PA_63_), 63 kDa heptamer is presented in panel (**A**). The PA monomer is comprised of four domains. A hydrophobic region implicated in LF and EF binding is exposed after furin cleavage in Domain I (blue), the *N-*terminus. Domain II (pink) is the heptamerization domain that contains a flexible loop thought to contribute to membrane insertion. The function of Domain III (yellow) is currently unknown, while Domain IV (purple) is the *C-*terminus, which comprises the receptor binding site (Image credit: R. John Collier, Harvard Medical School). Panel (**B**) is the Anthrax edema factor protein bound to calmodulin. The catalytic edema factor is represented in light blue while calmodulin is depicted in turquoise. Panel (**C**) is the Anthrax lethal factor (LF). Domains I-IV of LF are indicated as 1-4, respectively. A representative sequence of MAPKK-2 is located within the active site next to domain IV. Panel (**D**) contains a computational prediction of anthrax holotoxin structure. Both a side view (left) and top-down view are shown (right) (Image credit: T. Nguyen, National Cancer Institute [127]).

Although PA lacks enzymatic activity, it functions to facilitate entry of the LF and EF subunits into the host cell. The PA subunit is initially produced as an 83 kDa polypeptide (PA_83_) that binds to either of two identified anthrax receptors, tumor endothelial marker 8 (TEM8 or ANTXR1) or capillary morphogenesis 2 (CMG2 or ANTXR2), [113]. Upon binding to its receptor, PA is processed by the host endoprotease furin into a 63 kDa form (PA_63_) [114]. This processed form of PA is biologically active, and in conjunction with its receptor, self-associates into a heptameric pre-pore structure (PA_63_^7mer^) (Figure 6a). The water soluble heptamer is 85 Å high with a pre-pore average diameter of 160 Å, and a lumen average diameter of 35 Å [115]. The oligomerization of PA also induces the seven bound receptors to cluster in lipid rafts or detergent-resistant membrane microdomains implicated in toxin complex endocytosis [116]. The PA heptameric complex competitively binds up to three LF and/or EF subunits (Figure 6d) [117]. The binding of LF or EF to the pre-pore structure triggers activation of src-like kinases to initiate its uptake and induction of a conformational change in the PA heptamer that may later facilitate LF and EF translocation into the cytoplasm [118,119]. Once the receptor is activated, the anthrax complex is endocytosed via ubiquitin, actin, and clathrin dependent mechanisms and is then fused with an endosome [120]. Following toxin uptake, formation of a pore in the endosome bilayer is required for LF and EF transport into the cytoplasm. Translocation of LF and EF into the cytoplasm has been shown to be pH specific. This pH sensitivity is due to the protonation of His-121 and Glu-122 amino acid residues that permit disruption of a specific salt bridge. Disruption of the salt bridge results in decreased stability of PA and receptor interactions, leading to detachment of PA domain II, a peptide segment necessary for pore formation [121].This pore, which develops via protonation of negative amino acid residues, acts as a proton/protein symporter that drives translocation of LF and EF into the cell cytoplasm [122]. Before translocation, both LF and EF must be unfolded in order to fit through the lumen of the PA_63_^7mer^ pore, as shown by the presentation of barriers to unfolding, causing blockage of translocation [123]. Experiments have shown that the rate limiting step of LF translocation is in the unfolding of the amino-terminal beta-sheet subdomain catalyzed by the Phe-clamp active site of the PA_63_^7mer^ pore [124,125]. Translation of both LF and EF into the cytoplasm is initiated at the *N-*terminus [126]. Once in the cytoplasm, LF and EF exert their cytotoxic effects. 

Lethal Factor (LF) is a zinc-dependent metalloprotease that targets mitogen-activated protein kinase kinases (MAPKKs) [128,129]. The LF polypeptide contains four domains, the fourth of which contains the active site (Figure 6c) [130]. LF exhibits its toxic activity by specifically cleaving MAPKKs within the *N-*terminal proline-rich region that precedes the kinase domain. Protein cleavage specificity is achieved by recognition of a peptide consensus motif marked by a sequence of hydrophobic residues followed by basic residues [131]. Via cleavage, LF is able to disrupt MAPKK phosphorylation activity. Because MAPKKs are important in major signal transduction pathways, this disruption leads to a decrease in transcription and subsequent protein synthesis. 

Edema factor, the other catalytic subunit of anthrax toxin, acts as a calcium independent calmodulin-dependent adenylate cyclase that functions by increasing the intracellular concentration of cAMP [132,133]. Recent analysis of the crystal structure of EF bound to calmodulin (EF-CaM) reveals much about the activity of the enzyme (Figure 6b). Although structurally different from mammalian adenylate cyclases, EF-CaM uses a two-metal-ion catalysis reaction that is partially facilitated through a histidine, which initiates the deprotonation of ATP [132]. Together, with the metalloprotease activity of LF, the increased levels of cAMP resulting from EF-CaM activity produce a variety of cytotoxic effects that include altered water homeostasis, which significantly disturbs intracellular signaling pathways.

### 6.2. Immunological Activity and Clinical Applications of Anthrax

For *B*. *anthracis* to attack the host, anthrax toxin dismantles the first line of host defense, the innate immune system. Phagocyte chemotaxis is potently inhibited by the pertubation of chemokine receptor signaling [134]. Alternatively, EF greatly impairs neutrophil actin-based motility and also inhibits endothelial cell chemotaxis via indirect activation of Epac and Rap1 [135,136]. Previously shown by Kim and Bockoch, it is possible that anthrax EF inhibits Nox1 mediated reactive oxygen species (ROS) formation in gut epithelial cells, therefore circumventing an innate immune response in host epithelial cells [137]. Anthrax also has a similar effect on phagocytes. ROS and cytokine production necessary for macrophage function is inhibited by EF and LF, respectively, due to their dependence on the MAPK pathway [138]. Additionally, LF induces caspase dependent apoptosis of macrophages, which is aided by the circumvention of survival signaling cascades [139,140]. It is interesting to note that alveolar macrophages display a resistance to anthrax toxin action, most likely due to low ANTRX1 and ANTRX2 expression [141].

Dendritic cells (DCs) are antigen presenting cells that can be considered to be both an initial receptor for identifying pathogen attack and a bridge between innate and scalable adaptive immunity. Lethal factor impairs DC MHC antigen presentation via the ERK1/2 MAPK pathway [142]. In addition, LF causes a distinct downregulation of costimulatory factors CD80, CD86, and CD40, as well as down regulation of several inflammatory cytokines [143,144]. In the presence of EF, which is known to upregulate DC costimulatory factors, LF is still able to suppress DC activation. Dendritic cell maturation, as marked by CD83 synthesis, is induced by EF either alone or in combination with LF [145]. Suppression of the adaptive immune response is further exacerbated by the ability of EF to upregulate ANTRX1 mRNA expression and subsequently, protein availability [146]. The high expression of ANTRX1 receptor permits larger amounts of anthrax toxin to be taken up by DCs. A consequence of this mechanism is the initiation of caspase-3 dependent apoptosis of human DCs by LF [147].

The suppression of adaptive immunity by anthrax toxin is an essential part of *B*. *anthracis* evasion of the host immune response. Both LF and EF act directly on T lymphocytes by altering their immunogenic functions. In the presence of these anthrax toxin subunits, both proliferation and cytokine production of activated T cells are greatly inhibited [148]. Also, anthrax toxin disrupts T cell receptor initiated activation via the MAPK pathway. Further, MAPK dependent IL-2 production is also inhibited [149,150]. Due to their dependence on helper T cells, activation of B lymphocytes is blocked by anthrax toxin. In addition, anthrax toxin can directly affect B cell function by targeting the MAPK pathway. Thus, LF is able to potently inhibit B cell proliferation and antibody production [151].

The mechanism of host immune suppression employed by anthrax toxin opens the possibility of using its subunit, PA, as a safe and effective adjuvant. Similar to other AB toxins and their subunits, there is promise that PA may have the ability to act as both an anti-inflammatory or as a pro-inflammatory immunomodulator, depending on the antigen or autoantigen to which it may be conjugated. The recent demonstration, with an oral administration of PA conjugated to the poly-γ-glutamic acid (GPA) capsule peptide, shows that PA can be used as a vaccine to induce protection against lethal doses of anthrax toxin in guinea pigs, which lends further support to these notions [152]. Due to the controversy surrounding the toxin subunit’s ability to elicit contradictory responses, this field remains available for exploration. To realize the full potential of PA and other AB toxin subunits to serve as adjuvants for treatment of infectious and autoimmune diseases, a more complete investigation will be required. 

## 7. Ricin Toxin

### 7.1. Structure, Pathogenesis and Function

**Figure 7:**
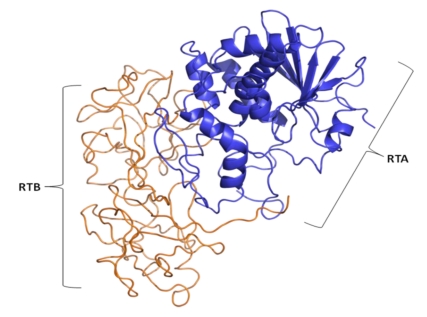
The crystal structure of ricin enterotoxin protein showing the A subunit (blue) and B subunit (gold) polypeptide chains. Ricin contains a catalytically active ribosome-inactivating 32 kDa A chain (RTA) linked by disulfide bonds to a galactose-binding lectin B subunit 34 kDa (RTB) [156].

Ricin enterotoxin is the prototypical lectin toxin. It is synthesized abundantly in the castor oil plant *(Ricinus communis)*.Additional plant species, including *Abrus precatorious* (Rosary pea), synthesize a lectin enterotoxin molecule (Abrin) almost identical in protein structure and action to ricin. Ricin enterotoxin exists in several isoforms, including ricin D, ricin E, and the closely related *ricinus communis agglutinin* (RCA) molecules [153,154]. Similar to Shiga toxin in its mode of action (Figure 7), ricin holotoxin contains a catalytically active ribosome-inactivating 32 kDa A chain (RTA) linked by several disulfide bonds to a galactose-binding lectin B subunit 34 kDa (RTB). In contrast to other bacterial AB toxins, the RTA holotoxin is a tetrameric toxin consisting of two separate ricin-like heterodimers containing only RCA subunits [155]. Thus, RCA is a strong hemaglutinin, but a rather weak toxin [155]. 

Ricin toxin is synthesized as a single polypeptide that is cleaved into both A and B subunits. The ricin toxic A subunit was discovered to be an *N-*glycosidase that depurinates specific adenine residues in rat 28S rRNA and in the 23S rRNA from *E*. *coli* [157]. Recent experimental evidence suggests ricin depurinates the eukaryotic ribosome by employing different docking sites on the ribosomal stalk. In *Saccharomyces cerevisiae*, surface Plasmon Resonance was used to show that the RTA subunit of ricin binds to the P1 and P2 proteins for its cytotoxicity [158]. The toxin does not, by itself, degrade RNA chains. However, depurination makes the RNA susceptible to hydrolysis at both an alkaline pH, and in an acidic environment [159]. As a result, the subunit is able to inactivate several thousand ribosomes faster than the cell can construct new ones [160]. In ricin as well as other type 1 ribosome inactivating proteins (RIP), a number of highly conserved residues, such as Glu177 and Arg 180, are important for enzymatic activity of the A subunit [161,162].

The galactose specific-lectin RTB subunit is responsible for binding ricin to both glycoprotein and glycolipids on the cell surface. The promiscuous binding of ricin to a wide variety of galactosidases and glycoproteins makes it difficult to identify specific ricin receptors. Also, it is known that ricin receptors are highly proteinaceous [163]. The lectin nature of ricin enhances cellular attachment and endocytosis of the toxin [164,165]. Experimental evidence has shown that several mechanisms of ricin endocytosis are cholesterol dependent [166]. The removal of cholesterol from the plasma membrane or the addition of cholesterol-binding drugs, such as filipin, results in the disappearance of caveolae and the inhibition of material uptake of clathrin pits [167].

One well characterized pathway leading from endosomes to the Golgi apparatus is the Rab9-dependent pathway from late endosomes to the Golgi apparatus [168]. Ricin transport was found to be independent of rab9, but sensitive to MβCD [169,170]. Ricin transport was also shown to occur via rab5 dependent vesicles [171]. Taken together, the experimental evidence shows that ricin may be transported by mechanisms similar to those used in the transport of cholera toxin and Shiga toxin from the cell surface to the trans-Golgi network. However, unlike cholera toxin, ricin trafficking from the trans-Golgi network to the ER remains independent of the KDEL motif. Interestingly, ricin can interact with calreticulin (which has a KDEL retention motif) in the Golgi network [172]. 

Most proteolytically cleaved toxins enter the ER with the A and B subunits linked by a disulfide bond. The ricin B subunit hinders the A subunit catalytic activity, thus inactivating holotoxin activity against free ribosomes. As a result, cleavage of the AB subunits is necessary for ricin mediated cytotoxicity [173,174]. The ER chaperone protein, disulfide isomerase (PDI), may be responsible for dissociating the A subunit from the B subunit, and may itself be reduced in the process by thioredoxin [175,176]. This reaction allows free RTA subunits to interact with lipids, inducing membrane instability [177].

### 7.2. Immunological and Clinical Applications of Ricin

Ricin is classically known for its strong ability to elicit an immune response. High titers of anti-ricin IgG antibodies were generated in mice challenged with sub-lethal doses of formalin-inactivated ricin toxoid [178,179,180,181,182]. Monoclonal and polyclonal anti-ricin antibodies have also been synthesized and were found to be protective against either the RTA or RTB subunits [179,183,184,185,186,187]. Vaccines against ricin are developed with caution, due to the possibility of generating antibodies that could potentially enhance cytotoxicity [185,187,188]. While toxoid vaccines have proven to be protective in animals, applications in humans may present further concerns [178,189,190,191,192]. Nonetheless, ricin has recently found increasingly widespread use as a research tool for the study of ribosome inactivation and protein transport. Coupled to ligands, ricin conjugates have been used to target and destroy tumor cells or tumor vasculature in specific cancer therapeutics [193,194].

In contrast to the well established property of ricin toxin as a strong inducer of immunity, the RTB subunit has shown increased promise for use as an enhancer of immune tolerance. When genetically linked to the *N-*terminus of insulin in *E*. *coli*, the bacterial synthesized INS-RTB fusion protein enhanced immunological suppression of pancreatic islet inflammation (insulitis), which is critical for prevention of Type 1 diabetes onset [76]. One potential caveat is the presence of substantial numbers of cysteine residues in the INS-RTB fusion protein, six cysteine residues in insulin and nine in RTB, which may substantially reduce the capacity of the fusion protein to fold into its native configuration. To obtain a correctly folded INS-RTB fusion protein for immunomodulatory studies, a gene encoding the INS-CTB fusion protein was transferred into potato plants to produce the natively folded fusion protein [195]. Plant protein expression systems frequently synthesize recombinant proteins that contain post-translational modifications useful for human immunotherapy.

To enhance subunit vaccine immune responses in the future, RTB fusion proteins may be used in combination, or sequentially, with other toxin B subunit-antigen or autoantigen fusion proteins. It is speculated that these prime boost multi-component immunization strategies will safely enhance pro-inflammatory or anti-inflammatory responses towards the antigen or autoantigen, respectively. Thus, development of safer and more effective B subunit based vaccines is a goal that is now receiving increased attention from the scientific community. 

## 8. Conclusions

The goal of this review was to examine the structure and function of prominent AB toxins and the implications of their properties to be used as adjuvant molecules for the enhancement of subunit vaccine efficacy. It has long been known that most subunit vaccines contain individual pathogen proteins, which have low inherent immunostimulatory properties. Thus, immunomodulatory molecules that can safely enhance vaccine-specific immunity are in increasing demand. Based on a growing awareness of their potential implications for subunit vaccine development, several issues remain to be addressed. These include clarification of the mechanisms underlying AB toxin immunomodulation and the ability to find response to challenges regarding the safety and efficacy of AB toxin subunit applications.

The molecular mechanisms responsible for adjuvant mediated immune responses remain largely obscure. Surprisingly, AB toxin subunits appear to elicit seemingly contradictory effects. This controversial observation further complicates the current understanding of how adjuvants may function. It is becoming increasingly apparent that the antigen or autoantigen, to which a toxin B subunit is fused, may determine whether the toxin B subunit serves as an adjuvant for increased immunity or for enhanced tolerance. 

Recently, the application of less immunogenic toxin B subunits that are fused with antigens to generate strong immunogenic properties has become an area of intense research. Further, the fusion of toxin B subunits with autoantigens, which stimulates, in general, immune tolerance against the linked autoantigen, has been found to exert a particularly useful effect in preventing the development of organ-specific autoimmune diseases [37,196,197,198]. On occasion, fusion of toxin B subunits to specific autoantigens, such as carcinoembryonic antigen (CEA) or prostate specific antigen (PSA), resulted in stimulation of protective inflammatory responses [72,199]. However, while this observation provides hope for effective protective and interventional therapy against these forms of cancer, our laboratory, as well as others, are currently working on the elucidation of early mechanisms underlying specific immune responses with adjuvant fusion proteins. Identification of pathogen receptors on antigen presenting cells, such as dendritic cell TLR’s with their ability to bridge innate and adaptive immune responses, has provided a unique opportunity for identification of pathways that can be targeted for development of non-toxic mucosal adjuvants. 

Unfortunately, the development of immunomodulatory molecule research has been unduly inhibited, due to frequent challenges about regulating adjuvant safety and efficacy. Safety concerns may arise from the potential for adjuvant molecules to overstimulate the immune system, resulting in unwanted or chronic inflammatory responses. These conditions may lead to disturbed immunological homeostasis, resulting in the onset of allergy or autoimmunity. Hence, only a few immuno-modulated mucosal vaccines have been approved for human application. Further, complications have been observed with the application of holotoxins, such as LT, in mucosal vaccination. Nasal administration of LT was linked to a rare adverse reaction, the appearance of Bell’s palsy. To circumvent such problems, new generations of altered LT mutant adjuvants, such as LTK63, a molecule with reduced ADP ribosylating activity, were constructed and engaged in human trials by Novartis Vaccines [200]. In addition, it was discovered that intranasal co-delivery of such mutant molecules, together with HIV or tuberculosis antigens, was also linked to transient nerve paralysis [201]. Thus, it follows that the route of delivery of the adjuvant molecule can also be an issue. Intranasal delivery as seen in the LTK63 human trials may not be the safest route of vaccine delivery, due to possible retrograde axonal transport of the vaccine after neuronal ganglioside binding [201]. Unsatisfactory results using the holotoxin and mutants of the holotoxin has stimulated a change in research focus toward use of the non-toxic LTB subunit in an effort to avoid adjuvant induced toxicity. Alternatively, other mucosal routes, such as transdermal or sublingual delivery, may be safer and require further exploration. Thus, development of safer and more effective mucosal adjuvants for human vaccination remains a high priority. We hope that the many opportunities for improvement in vaccination strategies presented by members of this intriguing AB toxin family will encourage collaboration and the flow of information among excited researchers.
